# Impact of hearing aid usage on the quality of life of the older adult in KwaZulu-Natal

**DOI:** 10.4102/sajcd.v73i1.1134

**Published:** 2026-02-28

**Authors:** Zahra Mansoor, Xoliswe Mchunu, Nkos’Kwanele Mthembu, Simangaliso B. Ngwenya, Thabile Thwala, Jessica Paken

**Affiliations:** 1 College of Health Sciences, University of KwaZulu-Natal, Durban, South Africa

**Keywords:** hearing aids, hearing loss, quality of life, presbycusis, older population

## Abstract

**Background:**

Despite the benefits of hearing aids for patients experiencing presbycusis, their adoption and consistent use in this population remain low.

**Objectives:**

This study explores older adults’ experiences with hearing aid use to better understand its perceived impact on the quality of life (QoL) and factors contributing to inconsistent use.

**Method:**

Adopting a phenomenological approach, semi-structured, in-person interviews were conducted with eight current hearing aid users, aged 67–84 years, presenting with varying degrees of hearing loss. Data were analysed using inductive thematic analysis.

**Results:**

The findings revealed four key themes with three sub-themes each, highlighting a perceived positive impact of hearing aid usage on participants’ QoL. Improved hearing enhanced communication with family and friends, reduced social isolation and fostered greater participation in social activities. Reduced anxiety and frustration enhanced emotional well-being. Challenges included initial resistance to hearing aids, adaptation difficulties and specific listening environment issues. Socioeconomic factors, such as transportation costs, posed significant barriers for individuals without medical aid coverage, limiting access to audiological services.

**Conclusion:**

Hearing aid use was perceived to enhance the QoL of older adults by improving social interactions, emotional well-being, cognition and listening comfort. However, uptake remains low because of resistance, adaptation challenges, technological difficulties and socioeconomic disparities. Addressing these barriers through patient education, improved service delivery, and research is essential to allow for an improvement in one’s QoL.

**Contribution:**

This study provides insights into understanding factors influencing hearing aid usage in older adults who may have unique experiences and challenges related to hearing health.

## Introduction

The World Health Organization (WHO, 2021) estimates that by 2050, over 700 million people will experience disabling hearing loss, with the majority being older adults. Presbycusis, that is, hearing loss related to ageing (Garefis, 2025), which, when untreated, has been closely associated with cognitive decline (Lin, 2013), depression, social isolation and a greater risk of dementia (Jayakody et al., 2022), ultimately impacting one’s quality of life. Quality of life is subjective and based on different individuals’ outlooks on the journey of life they have experienced for themselves. It is defined as a ‘dynamic, multi-level and complex concept, reflecting objective, subjective, macro-societal and micro-individual, positive and negative influences which interact together’ (Brown et al., 2004, p. 113).

Several studies (Moroe & Vazzana, 2019; Picou, 2020; Yamada et al., 2017; Ye et al., 2023) have demonstrated that hearing aid use significantly improves quality of life by enhancing emotional well-being, supporting cognitive health and reducing listening effort. By alleviating communication difficulties, hearing aids promote emotional stability, independence, vitality and social participation. Evidence also suggests that hearing aids may delay cognitive decline, with reported improvements in executive functioning, vitality and overall mental well-being, indicating a potential protective effect against cognitive ageing (Deal et al., 2018; Holman et al., 2022; Loughrey et al., 2018; Niazi et al., 2020; Zahl, 2023). In addition, hearing aid use has been shown to reduce listening fatigue and cognitive load by improving speech intelligibility and communication ease, thereby lessening exhaustion in daily interactions and facilitating sustained social engagement (Deal et al., 2018; Hyams et al., 2018; Monzani et al., 2022). Despite these benefits, uptake remains low, with 67% – 86% of adults with hearing loss not using their devices, often because of difficulties associated with advanced technology; however, these technological advancements may be beneficial for some (Moroe & Vazzana, 2019; Wirth, 2023).

In well-developed countries, audiological and other healthcare services are much more easily accessible in comparison to the South African context. In developing countries, service delivery is poor, resulting in individuals not using their hearing aids because they do not receive the necessary information regarding hearing aids, their maintenance and their benefits (Sooful et al., 2009). Factors contributing to reduced hearing aid usage include unfavourable word of mouth regarding hearing aids, and the need for multiple appointments with audiologists may be perceived as burdensome (Kochkin, 2017). A pilot study conducted in Johannesburg, South Africa, found that older adults encounter several difficulties when using hearing aids, including discomfort, a lack of knowledge about the devices, problems with maintenance and functionality, and a lack of patient involvement in the choice of devices (Moroe & Vazzana, 2019). Challenges like forgetfulness, cognitive decline and the expense of hearing aids have also been noted. Other barriers to adopting hearing aids include feelings of embarrassment (Eman, 2017), as there are still multiple misconceptions that assistive devices are cumbersome and unappealing.

The World Health Organization’s Quality of Life (WHOQOL) assessment provides a comprehensive framework for assessing the multifaceted impact of hearing aid usage on the elderly with hearing loss. The WHOQOL theory focuses on identifying underlying medical conditions, psychological and emotional implications and social interactions (Pomeroy et al., 2020). The WHOQOL tool is a generally accepted and comprehensive approach to assessing and understanding an individual’s quality of life across a variety of domains, including physical, psychological, social relationships and environmental health (Archuleta et al., 2021). This theory can help researchers understand feelings of frustration, loneliness, depression and worry, as well as the impact of environmental factors like noise, finances and spirituality on an individual’s sense of purpose and inner serenity (Pomeroy et al., 2020).

Utilising the WHOQOL, this study aims to explore how hearing aid use influences QoL among older adults with presbycusis, with particular attention to contextual barriers and facilitators relevant to the South African setting. Audiologists can therefore use the current study’s findings to illustrate to potential hearing aid users, who may be hesitant, the significance of hearing aids and how they can positively impact an individual’s quality of life.

## Research methods and design

### Research aim and objectives

This study aimed to explore the perceived impact of hearing aid use on older adults’ quality of life, including its influence on daily functioning, physical well-being, psychological and emotional health, social relationships and the broader environment.

### Study design

The study employed a qualitative research approach grounded in the principles of phenomenology. This method allowed for an in-depth exploration of participants’ experiences, particularly focusing on the daily events encountered by individuals (Chowdhury et al., 2020).

This design enabled the collection of rich, first-hand experiences to understand the lived experiences of hearing aid users in their everyday contexts.

### Study setting

The research was conducted in eThekwini, KwaZulu-Natal, South Africa. Participants were recruited from retirement homes and private practices, reflecting a mix of urban and peri-urban communities with diverse access to healthcare services.

### Study population and sampling strategy

The study employed purposive sampling. Eight participants (three males and five females) were interviewed. Participants were included in the study if they were 65 years of age and older, as this was the age at which individuals were classified as part of the geriatric population (Lohr, 2015). All participants had been diagnosed with hearing loss (unilateral or bilateral and ranging from mild to profound) by an audiologist to ensure uniformity of diagnosis. Furthermore, participants had been fitted with either air- or bone-conduction hearing aids, monaurally or binaurally, for a minimum of 1 year to ensure adequate acclimatisation.

Participants using cochlear implants, bone-anchored devices, or with diagnoses of mental illness, dementia, or Alzheimer’s disease were excluded to avoid heterogeneity and ensure accurate accounts of hearing aid impact.

Upon receipt of ethical clearance, recruitment of participants commenced. Recruitment involved requesting permission from the University of KwaZulu-Natal and private practice managers for posters to be displayed at these facilities. Assistance was also requested from private practice managers to distribute an e-poster on their social media platforms. Additionally, social media, such as Instagram, Facebook and WhatsApp, was used to circulate the poster to recruit potential participants. Once the respondents contacted the researchers, their eligibility was determined.

### Data collection

Data collection was conducted through the use of a semi-structured interview schedule developed based on questionnaires from literature (Gallagher & Woodside, 2018; WHO, 2012) and aligned to the objectives of the study, which were guided by the WHOQOL domains (Supplementary Material 1). All interviews were facilitated by a single researcher, as all participants spoke English and preferred to be interviewed in English, although a researcher was available to conduct the interviews in isiZulu if the need arose. In-person interviews were conducted and recorded via Voice Memos and the camera application using a mobile device (iPhone 12 Pro), facilitating accurate data capture while enabling the observation of non-verbal cues such as body language and facial expressions, which are crucial when interviewing participants with hearing loss (Jennings, 2005). All interviews were conducted in private rooms (in retirement homes, participant homes and boardrooms at the university) and were of the duration of 20 min – 30 min.

### Data analysis

The data were analysed using inductive thematic analysis, a data-driven approach that identifies and reports patterns directly from the dataset (Braun & Clarke, 2006; Castleberry & Nolen, 2018). All authors reviewed the data independently; thereafter, they combined and compared their findings to reach a consensus on the results (Golafshani, 2003). The six-step analysis process, developed by Braun and Clarke (2006), was followed in this study and included: (1) becoming familiar with the data, (2) generating coding categories, (3) generating themes, (4) reviewing the themes, (5) defining and naming the themes and (6) producing a comprehensive report with the assistance of the qualitative data analysis program, NVivo. Data analysis co-occurred with data collection, and no new themes emerged following the analysis of the eighth interview, indicating data saturation had occurred.

### Trustworthiness and credibility

All interviews were facilitated by the same researcher, which ensured that the manner in which the semi-structured interview questions were asked was consistent. Member checking was also conducted by sharing the transcript of the participants’ interview with them and querying its accuracy to maintain credibility (Stahl & King, 2020). During the research process, the researchers reflected on their prejudices and were open and honest about the potential impact of their experiences and viewpoints on the research.

To ensure content validity, the interview schedule was created by adapting and reviewing relevant literature (Gallagher & Woodside, 2018; WHO, 2012) and obtaining information from participants who strongly met the inclusion and exclusion criteria. The pilot study was conducted with two participants; the data obtained from these interviews were analysed to assess whether the questions in the interview schedule adequately addressed the study’s objectives and were appropriate, straightforward and understandable to the participants. Results obtained in the pilot study were not included in the main study.

### Ethical considerations

An application for full ethical approval was made to the University of KwaZulu-Natal Humanities and Social Sciences Research Ethics Committee on 25 May 2024. The ethics approval number is HSSREC/00006775/2024. Furthermore, informed written consent was obtained from all participants, and participants were made aware that they can withdraw from the study at any point without any repercussions.

## Results and discussion

### Description of participants

Eight participants, three males and five females, between the ages of 67 years and 84 years, participated in this study. A brief description of the participants is provided in Table 1.

**TABLE 1:** Description of participants.

Pseudonyms	Age (years)	Residence	Degree of hearing loss	Hearing aid type	Hearing aid experience (years)
Mary	74	Retirement home	Unilateral, mild to moderate (right ear)	Behind-the-ear (BTE)	Over 1
Grace	73	Retirement home	Bilateral, moderate	Monaural BTE (left ear)	Approximately 2
John	76	Lives alone	Bilateral, mild to severe	Binaural BTEs	15
Teddy	78	Unavailable	Bilateral, mild to profound	Binaural BTEs	6
Jane	80	Old-age home	Bilateral, mild to severe mixed	Binaural hearing aids	35
Lydia	67	Unavailable	Bilateral, mild to severe	Monaural BTE	6
James	70	Unavailable	Bilateral, mild to profound	Monaural BTE	Approximately 5
Eunice	84	Old-age home	Bilateral, moderate to severe	Monaural BTE	Approximately 11

**FIGURE 1:**
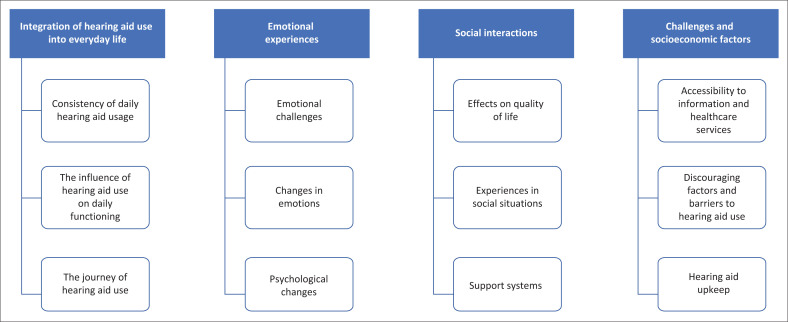
Diagram illustrating emergent themes.

Inductive thematic analysis revealed four key themes, namely integration of hearing aid use into everyday life, emotional experiences, social interactions and challenges and socioeconomic factors with their relevant sub-themes as indicated in Figure 1.

### Theme 1: Integration of hearing aid use into everyday life

#### Subtheme 1.1: Consistency of daily hearing aid usage

In the present study, participants reported higher rates of consistent hearing aid use among participants, with five of the eight participants wearing them from morning to bedtime, which aligns with the study conducted by Dillon et al. (2020), who found improved usage among hearing aid users. However, some participants still reported inconsistent use, below the recommended 6 h – 8 h (Flynn et al., 2022), as seen in the responses by the following participants:

‘Not as much as I should, so on an average, probably five hours a day.’ (James)

‘When I watch TV, which is like for 3 to 4 hours, then days where nothing is happening, I don’t wear them when I go to … not exactly a religious person, but I go to service, and so you have to listen to the man who’s giving the talk I wear them.’ (Teddy)

Participants’ consistent use of their hearing aids was attributed to the comfort and functionality of the devices, which facilitated positive auditory and social experiences. In contrast, barriers to optimal use included discomfort, situational preferences, poor sound quality and a lack of perceived benefits:

‘Well, I’ve got a problem. They said the right-hand side is the bad ear. Yeah, this one was going. So, they gave me hearing aid for this. Yeah, okay and I’ve been back five times with this one, and it still is not right. So, I’m lopsided. Do you hear what I’m saying? It is very frustrating because I hear someone talking to the one side and I look there.’ (Grace)

These challenges may stem from insufficient counselling and education during the fitting process, or a lack of ongoing support from audiologists and family members. Without appropriate follow-up care, some users struggled to integrate hearing aids into their daily routines, potentially missing out on broader benefits such as improved emotional well-being and communication abilities.

#### Subtheme 1.2: The influence of hearing aid use on daily functioning

Participants reported an improvement in their daily lives through the use of hearing aids, particularly in the perception of environmental sounds, enhanced clarity in communication and increased satisfaction with their auditory experiences. The responses from the study participants reflect several key benefits that align with the findings of Avierinos et al. (2024) regarding meaningful life changes following hearing aid use, as seen by one participant’s response:

‘Well, when I do wear them, it has improved a lot. I can even hear birds singing that I didn’t know before, and you can hear leaves rustling on the ground, which I don’t hear any other time. So, it’s little things like this that I can hear, which if I haven’t got them on, I don’t hear them as soon as I put them on, I hear them.’ (Mary)

Improved hearing reduces communication difficulties, significantly enhancing social experiences and positively impacting an individual’s lifestyle. When communication barriers are minimised, individuals feel more confident and comfortable engaging in conversations, whether at family gatherings, social events or in professional settings. This increased clarity fosters deeper connections, reduces feelings of isolation and creates a more fulfilling lifestyle.

These findings support those of Avierinos et al. (2024) and Petersen et al. (2022), who noted that hearing aids enhance communication by reducing background noise, improving speech comprehension and making communication easier. Similarly, Hyams et al. (2018) reported that hearing aids reduce listening fatigue and improve speech intelligibility, contributing to better daily functioning.

#### Subtheme 1.3: The journey of hearing aid use

The journey of hearing aid use among older adults is marked by diverse experiences, shaped by individual adaptation, device characteristics, and the level of support received. Initial challenges are frequently reported by first-time users (Oosthuizen et al., 2022), often because of the sudden amplification of environmental sounds:

‘Well, in the beginning, everything was very noisy as far as I was concerned … noisy, but I’ve got used to it now.’ (Mary)

This illustrates a common pattern in which early difficulties ease with continued use and adaptation.

For others, however, persistent challenges undermine consistent use. One participant highlighted frustrations with in-the-ear (ITE) devices, noting that difficulties with insertion led her to abandon one set altogether:

‘You can’t even get it in. So, I neglected that … when it doesn’t go in, you just want to leave it.’ (Jane)

These frustrations align with findings that elderly individuals often struggle with ITE devices because of age-related declines in dexterity (Jorbonyan et al., 2021).

While some studies report higher satisfaction with ITE hearing aids (Dell’ Antônia et al., 2013), alternative styles such as behind-the-ear (BTE) or receiver-in-canal (RIC) devices may be more suitable for older users, given their larger components and easier handling (Heliopoulos, 2021).

Experiences also differ depending on fitting strategies and the degree of hearing loss. Another participant that was fitted unilaterally despite asymmetrical hearing loss, expressed frustration at the imbalance:

‘So, they gave me a hearing aid for this [*left ear*] … and I’ve been back five times with this one, and it still is not right. So, I’m lopsided.’ (Grace)

Her dissatisfaction reflects evidence that unilateral fittings in cases of asymmetrical hearing loss worsen auditory challenges and heighten the risk of non-use (Frosolini et al., 2023; Noh & Lee, 2023). Research further indicates that bilateral fittings offer superior auditory and quality-of-life outcomes, particularly in cases of asymmetry (Hoppe et al., 2022; Orji et al., 2020).

Underlying these varied journeys is the role of personal motivation. Moore et al. (2021) emphasise that highly motivated individuals are more likely to persist with their devices despite difficulties, whereas those with lower motivation often discontinue use because of discomfort or lack of perceived benefit (Franks & Timmer, 2023). Overall, the hearing aid journey is highly individualised, ranging from smooth adaptation, as in Mary’s case, to persistent difficulties, as seen with Jane and Grace. These experiences highlight the importance of careful device selection, bilateral fitting where appropriate, and the provision of ongoing audiological support to address physical, perceptual and motivational barriers to long-term hearing aid use.

The theme of integration of hearing aid use in everyday life primarily aligns with the physical health domain of the WHOQOL framework, as the effectiveness of hearing aids directly affects an individual’s physical well-being and ability to participate in daily activities. It also intersects with the psychological health domain, given that adapting to hearing aids can impact self-image and mental state. Emotional responses, such as frustration or relief during the adjustment period, are closely tied to this domain and can influence overall quality of life.

### Theme 2: Emotional experiences address objectives two and three

#### Subtheme 2.1: Emotional challenges

Untreated hearing loss is strongly associated with higher rates of depression and anxiety (Sharma et al., 2021). The stigma surrounding hearing loss further contributes to these negative emotions. In the current study, most participants reported similar emotional struggles related to their hearing impairment, reinforcing the link between hearing loss and mental well-being.

The following statements were made by the participants when asked about the effect hearing aid use had on their emotional well-being:

‘Very frustrating. I’ve got a complex about it.’ (Grace)

‘I get so worried.’ (Eunice)

Grace’s experience highlights the challenges of adjusting to hearing aids, particularly for those with little prior knowledge or experience. Grace’s use of the term ‘complex’ suggests an ongoing internal conflict and self-consciousness about using hearing aids, which can lead to diminished self-esteem and increased emotional distress (Humes et al., 2017). These findings align with Timmer et al. (2023), who noted that the adaptation process can contribute to isolation and social withdrawal because of perceived stigma or difficulty using the device effectively. Similarly, Eunice’s feeling of worry underscores the broader anxiety surrounding the reliance on a hearing aid and the fear of its inadequacies, reflecting the complex emotional and psychological challenges accompanying the transition to using such a device (Ferguson et al., 2020). These emotional responses highlight the need for ongoing support and counselling to help individuals manage the emotional burden associated with hearing loss and hearing aid use.

While some participants expressed significant emotional distress, frustration and anxiety related to hearing aids, some participants reported no emotional challenges related to hearing aid use:

‘I didn’t have nothing [*no emotional challenges*], only family trauma.’ (Jane)

‘Absolutely none.’ (Lydia)

The above responses suggest that the emotional response to hearing aids can vary widely, which aligns with research by Ferguson et al. (2020), who found that emotional responses to hearing aids can be influenced by personal resilience, social support and individual coping strategies.

Social support is essential in reducing stress and enhancing satisfaction with hearing aid use.

Encouragement from family, friends and support groups provides both practical assistance and emotional reassurance, easing the adjustment process (Singh et al., 2015). Effective communication and empathy help mitigate feelings of isolation, emphasising the need for a family-centred approach. However, the extent and nature of support may differ across cultural contexts. In South Africa, traditional family structures play a vital role in caregiving and well-being (Gambe et al., 2023). Involving family fosters better communication, reduces stigma and eases emotional distress, which enhances audiological services, particularly in areas with limited access to professional services (Swanepoel & Hall, 2010).

#### Subtheme 2.2: Changes in emotions

The responses obtained from the study collectively suggest that for some individuals, the emotional impact of hearing aid use may be neutral or minimal, which could be influenced by factors such as prior adaptation to hearing loss, low emotional expectations or satisfaction with the device meeting basic functional needs. Some individuals may not report a strong emotional response, particularly if their primary expectation was functional improvement rather than emotional relief. Similarly, Kochkin (2017) highlighted that emotional satisfaction derived from hearing aids is inconsistent and is influenced by individual differences in the perceived value of emotional well-being. However, one participant expressed ongoing frustration despite using hearing aids, stating:

‘You know the weird frustration again because I can only hear from properly on the one side and the other side. If I answer the phone, I’ll take the right-hand hearing aid out and put speaker on the phone. Believe it or not. Because I can’t hear anything.’ (Grace)

This response highlights a common challenge faced by some hearing aid users: Hearing aids enhance general hearing but may be less effective in complex auditory settings, such as telephone conversations, leading to frustration (McCormack & Fortnum, 2013). Users often struggle in challenging listening environments, which can reduce satisfaction (Wong et al., 2003), which can also affect social interactions, as misunderstandings in speech comprehension may decrease engagement (Palmer et al., 2016). Hearing aid users often face challenges in complex environments because of the device’s inability to manage background noise and reverberation, which makes it difficult to identify speech signals (Xia et al., 2018). Moreover, the effectiveness of hearing aids varies between users, as individuals with different types and degrees of hearing loss may experience inconsistent benefits, leading to frustration and dissatisfaction (Bannon et al., 2023).

Additionally, misalignment between users’ expectations and the performance of their hearing aids can lead to disappointment, especially in complex listening environments (Meyer et al., 2014). Audiologists play a crucial role in setting realistic expectations and providing clear information on the capabilities and limitations of hearing aids. Personalised fittings, fine-tuning and regular follow-ups are vital for optimising hearing aid performance and user satisfaction, particularly in social interactions and emotional well-being (Choi & Toma, 2014).

Audiologists should also provide counselling and education, particularly for challenging environments, and address the emotional and psychological impacts of hearing aid use, offering additional resources when necessary (Van Wilderode et al., 2023), which helps to minimise disappointment and improve overall satisfaction with hearing aids.

In contrast, another participant reported significant improvement and satisfaction with hearing aid use, stating:

‘I feel much better, and I feel it. You know, the hearing aid is a fantastic aid for people to adjust, and for me, it made a very big difference.’ (John)

John’s experience aligns with Chisolm et al. (2007), who found that many hearing aid users report improved emotional stability and confidence in social interactions. Additionally, Jang et al. (2024) highlighted that hearing aids enhance users’ perception of their hearing abilities, leading to greater satisfaction and better adjustment to hearing loss. When users perceive that their hearing aids effectively address their needs, they experience increased satisfaction and a more positive outlook on life, contributing to better emotional adjustment (Holman et al., 2022).

#### Subtheme 2.3: Psychological changes

There is limited evidence of psychological changes among hearing aid users; however, some experiences suggest subtle shifts. Grace shared an experience hinting at emotional effects, aligning with studies that highlight stigma-related feelings, such as self-consciousness or embarrassment, associated with hearing aid use (Wallhagen, 2009). Stachler et al. (2012) similarly reported that while hearing aids enhance hearing ability, societal perceptions can lead to psychological effects like anxiety and embarrassment:

‘I used to wear my hair very short, and since I’ve had the hearing aid, I’m wearing it longer.’ (Grace)

Grace’s decision to grow her hair longer may reflect a desire to adapt her appearance to better incorporate the hearing aid, possibly to make it less noticeable or to feel more comfortable with her self-image. This minor change indicates that hearing aids can influence aspects of personal identity and self-esteem.

### Theme 3: Social interactions addressed objectives one and three

#### Subtheme 3.1: The impact on older adults’ lifestyle

Older adults, particularly those over 65, often experience multiple medical conditions that significantly impact their quality of life (Vu et al., 2019). The presence of comorbidities, along with hearing loss, can lead to physical and cognitive challenges, further affecting daily functioning. However, research has highlighted the substantial indirect benefits of hearing aid use in this population (Tognola et al., 2019; Yadav et al., 2023; Zhang et al., 2024). Studies have shown that regular and consistent use of hearing aids leads to notable improvements in quality of life (Hyams et al., 2018; Picou, 2020; Yamada et al., 2017), and is reiterated by a participant reporting:

‘It has impacted me quite tremendously, for the better.’ (John)

The findings of this study align with those of Zafar et al. (2021), confirming that consistent hearing aid use enhances daily living and personal well-being while also having broader socio-economic benefits. On the other hand, one mentioned:

‘I can hear better, but I think it’s impacted other people better.’ (James)

James’s remarks suggest that his improved hearing and lifestyle have positively impacted those around him, including family and friends. However, while he has noted some benefits, no significant changes have been reported. This may be *related* to the severity of his hearing loss, which ranges from mild to profound. Factors such as hearing loss severity and dissatisfaction with hearing aids can influence their effectiveness in improving quality of life (Dell’ Antônia et al., 2013), particularly for individuals with more advanced hearing impairment.

#### Subtheme 3.2: Experiences in social situations

The study findings indicate that hearing aid users experience significant improvements in social interactions, leading to more inclusive and fulfilling social experiences. Enhanced hearing and communication abilities have allowed them to engage more confidently in social settings, fostering a sense of belonging and participation. As a result, they can enjoy richer and more meaningful connections with others, which aligns with the findings of Yamada et al. (2017).

One of the participants’ response regarding the impact of hearing aid use on their social interactions and relationships with others can be noted as follows:

‘I think it’s helped me because in a social situation where you’re sitting with more than two people, if I don’t hear something, I don’t have to keep saying sorry or excuse me, so it’s helped me to be more involved in that conversation.’ (Lydia)

However, another participant reported a challenge in social situations despite using hearing aids and mentioned:

‘It’s still a little bit, you know, it’s not the natural hearing, you know. So, I’m always conscious that I’ve got my hearing aids on. Even with the hearing aid, there is challenges sometimes; you can’t hear the people properly. And sometimes you must really strain because a lot of people they talk very softly and if they don’t talk loud and bold, it’s difficult to pick it up, you know.’ (John)

John’s concern regarding the inability of hearing aids to replicate natural hearing is a frequently reported issue among users. A study by Avierinos et al. (2024) corroborates this difficulty, noting that 41 of their 653 participants experienced problems with hearing aid use, specifically regarding the device’s failure to replicate natural hearing. While hearing aids incorporate advanced technologies like multi-band broad dynamic range compression, they cannot fully replicate the function of the outer hair cells in the cochlea, as the intact auditory system operates with a complexity that current hearing aid technology is unable to fully replicate (Lesica, 2018).

#### Subtheme 3.3: Support systems

A support system (family, friends and healthcare workers) plays a vital role in hearing aid adoption and continued use. Thorén et al. (2013) suggested that individuals in retirement homes often experience a lower quality of life because of inadequate support systems. However, the findings of the current study indicate otherwise. Four of the eight participants interviewed reside in retirement homes, and the following responses were provided by participants when asked what their support system consisted of during the process of hearing aid adoption:

‘Family encouragement, yes.’ (Grace)

‘Well, I would say the hospital, the people at King Edward and here they were very good. I don’t have a complaint.’ (Mary)

The presence of robust support systems was noted, and the remaining participants reported similar sentiments, reflecting a broad consensus on the availability of support in their environments. Financial support is a crucial factor in hearing aid adoption, especially because of the high initial cost. Medical aid plays a pivotal role in alleviating the financial burden associated with purchasing and maintaining hearing aids, helping to alleviate stress (Kochkin, 2007), enabling individuals to use their hearing aids consistently without financial concerns. This was seen in the following response:

‘Well, as I said, I am an ex-policeman, so I have medical aid, and I have no problems. I just go to the doctors, and they give me a referral letter. I go to the audiologist, they do a complete analysis, and then, they apply for the hearing aids.’ (John)

Social interactions fall under the social relationships domain within the WHOQOL framework, which assesses personal connections and social support, both of which can be affected by hearing loss and hearing aid use. In this study, hearing aids facilitated effective communication, enhancing participants’ social interactions and improving their social lives and relationships, thereby positively contributing to overall quality of life. The study highlights how hearing aids support relationship building, participation in social activities and a sense of inclusion, essential elements of social well-being.

### Theme 4: Challenges and socioeconomic factors address objectives one and four

#### Subtheme 4.1: Accessibility to information and healthcare services

Easy access to healthcare services is crucial for managing hearing loss in older adults. Reliable access positively influences their engagement with healthcare, enhancing auditory health, social interactions and overall quality of life (Contrera et al., 2016; Liao et al., 2023). Gaffney and Hamiduzzaman (2022) highlight that the availability of appointments, support and health information significantly impacts healthcare participation. Additionally, older adults prefer face-to-face interactions with healthcare providers, as person-centred communication improves engagement and satisfaction (Murdin et al., 2022). Therefore, adapting healthcare systems to patients’ needs is essential for effective care.

Despite the positive reports of accessibility, challenges remain, particularly regarding communication barriers and health literacy (Gaffney & Hamiduzzaman, 2022). Difficulties in understanding health information and navigating healthcare systems can limit their ability to seek assistance effectively. Additionally, socioeconomic factors play a significant role in access, as individuals from disadvantaged backgrounds may struggle with transportation costs, restricting their use of available services (Mtimkulu & Khoza-Shangase, 2024). As one participant reported:

‘It was easier before, but now it is difficult because of transport.’ (Eunice)

Therefore, while the participants in this study reported ease of access, addressing these challenges through ongoing support and education is necessary to help older adults fully utilise healthcare services while also minimising unnecessary audiology consultations that could further increase financial burdens.

#### Subtheme 4.2: Discouraging factors and barriers to hearing aid use

Participants in the current study reported that they did not encounter barriers when obtaining their hearing aids because of medical aid coverage. However, one participant, indicated that affordability was a challenge in the acquisition process by reporting:

‘It was basically the affordability of the thing (hearing aids) that was a barrier.’ (Teddy)

While many older adults incur high costs for hearing aids, those with medical aid may have some of these expenses covered, as the extent of coverage varies, and not all medical aids fully cover hearing aids, which can still leave some financial strain on users. Participants with medical aid often have improved access to audiological services, which can boost user confidence and promote consistent use of hearing aids. In contrast, the study by Gallagher and Woodside (2018) highlights that many older adults face barriers because of insufficient access to audiological care.

However, the implementation of National Health Insurance (NHI) in South Africa aims to provide equitable healthcare access, including for hearing aids, particularly benefiting older adults without medical aid coverage (Ndlovu et al., 2022). The NHI could eliminate the financial barriers typically associated with private medical aid, potentially increasing hearing aid usage among those previously unable to afford them. By standardising procedures for obtaining hearing aids, the NHI could simplify the process, reduce confusion and encourage more older adults to seek intervention for hearing loss. The lack of barriers among older adults using medical aid in eThekwini highlights the importance of financial support, education and a strong healthcare system. As the NHI develops, it could improve access for all older adults, enhancing their quality of life through better hearing health and addressing challenges for those without medical aid to ensure equitable healthcare outcomes (Danemayer et al., 2022).

With half of the participants reporting that there was nothing discouraging them from adopting hearing aids, the remaining participants were discouraged by physical factors and environmental factors, as indicated by the following responses:

‘Nothing with the current HA [*hearing aid*]. Now the ITE HA [*in-the-ear hearing aid*], I neglected it because I couldn’t fit it in properly.’ (Jane)

‘Just knowing that I’m going to be sweating or that I’m going to be in a situation where I could get water on these devices, as they are expensive.’ (James)

Many studies highlight that stigma surrounding hearing loss and hearing aid use can discourage individuals from seeking help (Zheng et al., 2022). However, in the current study, participants appeared to have a positive self-perception regarding their hearing loss and the use of hearing aids, suggesting a cultural or community norm that supports their use. None of the participants reported stigma, possibly because of supportive family structures or community programs encouraging hearing aid use. This contrasts with other populations where structural barriers, such as limited transport, family support and availability of hearing care services, are prevalent (Reddy et al., 2019).

The participants in the current study reported a lack of barriers, which raises questions about the unique context of eThekwini and the specific experiences of its older population. The absence of barriers among participants with medical aid underscores the potential for improved quality of life through effective hearing aid use. Unlike other studies highlighting a lack of information as a barrier, this study sample might have received adequate information, leading to a more proactive approach to managing their hearing loss (Gallagher & Woodside, 2018).

#### Subtheme 4.3: Hearing aid upkeep

The findings of the current study contrast with previous research that highlights multiple financial and logistical barriers to hearing aid maintenance, such as repair costs, professional servicing and high initial purchase prices (Marcos-Alonso et al., 2023). This contrast may be explained by the fact that the study participants were pensioners with medical aid coverage residing in the eThekwini district, which may not be representative of the broader older population.

Access to follow-up services was reported to be relatively easy, likely because of proximity to hospitals or private audiology practices and the financial support provided by medical aid. As a result, concerns about follow-up costs were minimised, making battery costs the primary financial consideration for these individuals, as reported by the following participants:

‘Batteries is the only cost.’ (Teddy)

‘Only the purchase of the batteries. The service on the hearing aids is 2 years. So, I don’t pay for anything.’ (John)

The emphasis on battery replacement as the primary upkeep cost suggests that participants may be using user-friendly, low-maintenance hearing aids. Advances in modern hearing aid technology have improved durability and ease of use, contributing to greater user satisfaction and adherence (Kaur et al., 2020). Hearing aid upkeep involves various costs beyond batteries, including the initial purchase, fitting fees, maintenance, replacement parts and accessories. For older adults with medical aid, coverage can help reduce or eliminate expenses for repairs, adjustments and professional services, making hearing aid use more accessible and sustainable. Additionally, medical aid beneficiaries often consult audiologists more frequently, receiving thorough counselling during the fitting process. This promotes better hearing aid care, reduces maintenance issues and alleviates financial burdens, encouraging consistent use and improving hearing health outcomes.

**FIGURE 2:**
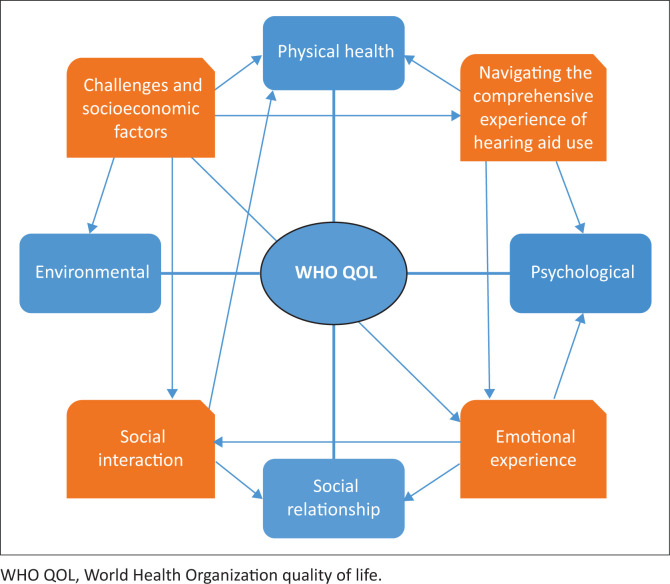
Alignment of themes with World Health Organization quality of life domains.

The theme of challenges and socioeconomic factors aligns with the environmental health domain, which includes financial resources and access to healthcare services. The study showed that socioeconomic status influences access to hearing aids and related services, with most participants reporting access to medical aid. Additionally, socioeconomic difficulties can impact emotional well-being and social participation, reflecting the interconnectedness of these quality-of-life domains, as illustrated in Figure 2.

By utilising the WHOQOL domains in the development of the objectives of the study and consequently the survey, this study offers a structured framework for exploring the diverse benefits of hearing aids, while also underscoring the importance of personalised interventions that address the comprehensive needs of older adults, with the ultimate goal of enhancing their overall quality of life (Govender & De Jongh, 2021). While the strength of the study can be observed by the stringent methodology, the inclusion of participants from diverse genders, ages and cultural backgrounds also allowed for a wide range of experiences to be captured, further enriching the findings of the study. However, limitations of the study include issues with the recruitment of participants. Although social media was utilised as a recruitment method, some older adults may have been missed, despite evidence of increasing social media usage among this demographic (Cotten et al., 2021). Additionally, limiting the study to eThekwini, KwaZulu-Natal, restricted the participant pool to this urban municipality, making the findings not generalisable to other regions or provinces of South Africa.

## Conclusion

Participants reported notable improvements in overall quality of life following hearing aid use, particularly with regard to auditory perception, daily functioning and social relationships. Although psychological well-being demonstrated minimal change, enhanced communication and reduced social isolation facilitated greater engagement in social activities. However, some experienced anxiety and frustration during the adaptation process, primarily related to device performance. Financial support, notably through medical aid, substantially alleviated costs associated with hearing aids, including fittings and follow-up appointments, thereby supporting consistent use despite ongoing expenses such as battery replacement. Therefore, the findings emphasise the need for National Health Insurance to improve access to hearing aids and associated services for eligible individuals, and the necessity of a patient-centred adoption process encompassing education, counselling and ongoing professional support.
